# An Image Enhancement Algorithm Based on Fractional-Order Phase Stretch Transform and Relative Total Variation

**DOI:** 10.1155/2021/8818331

**Published:** 2021-01-13

**Authors:** Wei Wang, Ying Jia, Qiming Wang, Pengfei Xu

**Affiliations:** ^1^School of Information Engineering, Pingdingshan University, Pingdingshan, Henan, China; ^2^Henan Hygiene and Health Cadre College, Zhengzhou, Henan, China

## Abstract

The main purpose of image enhancement technology is to improve the quality of the image to better assist those activities of daily life that are widely dependent on it like healthcare, industries, education, and surveillance. Due to the influence of complex environments, there are risks of insufficient detail and low contrast in some images. Existing enhancement algorithms are prone to overexposure and improper detail processing. This paper attempts to improve the treatment effect of Phase Stretch Transform (PST) on the information of low and medium frequencies. For this purpose, an image enhancement algorithm on the basis of fractional-order PST and relative total variation (FOPSTRTV) is developed to address the task. In this algorithm, the noise in the original image is removed by low-pass filtering, the edges of images are extracted by fractional-order PST, and then the images are fused with extracted edges through RTV. Finally, extensive experiments were used to verify the effect of the proposed algorithm with different datasets.

## 1. Introduction

Image sharpening means enhancing, that is, highlighting edges and texture features, while suppressing those unimportant planner areas, planner areas, and parts with constant grey levels. In addition, the original image is fused with enhanced edge and texture images (the simplest fusion is a pixel-by-pixel weighted average) to ensure a smooth transition between its grey levels. Therefore, in the enhanced image, the edges and key textures are highlighted, and other areas are also sharpened.

In the process of image enhancement, the causes of image degradation have not been analyzed, and the processed image is not necessarily close to the original image. In the literature [1], the K-means clustering algorithm was used to divide the image into several grey-scale intervals and then equate them separately. This algorithm has a better-enhanced effect on the images for grayscale distribution at both ends and more pixels in the grey-scale regions. Literature [2] proposed an algorithm to enhance the adaptive image based on a double histogram equation, which has to keep the average brightness of the output image close to the original image and avoid the tendency to magnify. Literature [3] proposed an image enhancement algorithm based on wavelet analysis and Retinex algorithm. Wavelet transform decomposes the image into multiscale images, removes noise from images with different frequencies, and uses a Retinex algorithm to enhance image details. Therefore, the combination of the two methods can improve the overall visual effect of the image and better highlight the details of the image. In the literature [4, 5], a better enhancement effect was achieved by enhancing the image contrast and the singular matrix of the image defined in the wavelet domain. In [6], an improved algorithm is proposed, which combines the adaptive total variation model and the impact filter model. Literature [7] used the minimum error method to automatically calculate the segmentation point of the piecewise linear grey-scale transformation, which improved the visual effect of the image. In [8], the improved histogram equalization method and the contrast enhancement algorithm were combined to improve the local information contrast enhancement of low illumination images. An adaptive smoothing and enhancement method for images was proposed in [9]; when filtering, the filter weights of the processed pixels are dynamically determined; after smoothing the inside of the image region, the edge of the region in the image is also sharpened and enhanced, which effectively solves the contradiction between image smoothing and enhancement.

The above literature all involves the image enhancement algorithms based on time domain and frequency domain variation. But it has been rarely found in domestic and foreign literature about the image enhancement for phase information changes. In 2015, M.H. Asghari and B. Jalali proposed a digital image transformation inspired by physical phenomena, called Phase Stretch Transform (PST). This paper attempts to improve the treatment effect of the PST on the information of low and medium frequency.

## 2. Relate Work

### 2.1. Basic PST

In 2015, M.H. Asghari and B. Jalali proposed a digital image transformation inspired by physical phenomena, called Phase Stretch Transform, which can simulate the propagation process of electromagnetic waves in the diffraction medium with the warping dispersive dielectric function. This method using phase-detection edges can simulate the diffraction process with an all-pass phase filter having a specific frequency and divergence dependency. The output phase prototype of the filter shows the change in image intensity value in the spatial domain, and the edge detection can be achieved after the thresholding and morphological processing of the phase prototype.

**Figure 1:**
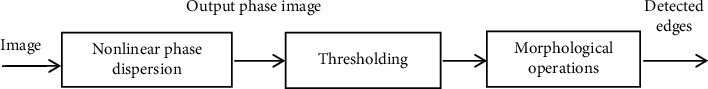
Schematic diagram of image edge detection process based on PST.

Figure 1 shows the process of digital image edge detection based on PST. A local filter kernel function first smoothed the original image, and then the phase operation of the nonlinear frequency function was performed in the frequency domain, called PST. Finally, edge detection was achieved through postprocessing such as thresholding and morphological filtering.

The mathematical model of PST in the frequency domain is

(1) $$\begin{array}{c} A\left(m,n\right)=∠IFFT2\left\{\tilde{K}\left(u,v\right)\cdot \tilde{L}\left(u,v\right)\cdot FFT2\left[B\left(m,n\right)\right]\right\}, \end{array}$$

where *A*(*m*, *n*) is the angle image, “∠” is an angle operation, *B*(*m*, *n*) is an original input image, FFT2 and IFFT2 are a two-dimensional fast Fourier transform and an inverse transform, respectively, and (u, v) is the frequency variable. $\tilde{L}\left(u,v\right)$ is the frequency response of the local smoothing low-pass filter, $\tilde{K}\left(u,v\right)=e^{j\phi \left(u,v\right)}$ is a frequency-dependent nonlinear phase warping kernel function, and *ϕ*(*u*, *v*) is a nonlinear function of the frequency variable.

Although any phase kernel function can be considered in the PST, the derivative of the kernel phase function *ϕ*(*u*, *v*) is a linear or sublinear function of the frequency variable according to the results of the literature [10]. For the phase kernel function prototype, a simple example is the inverse-tangent function of the “S” type. For the sake of simplicity, if it is further required that the phase warping operation is isotropic in the plain of the frequency domain, the degree of warping is only related to the polar radius *r* in the planar polar coordinate system of the o-uv frequency, but independent of the polar angle *θ*, that is, assuming that the nuclear phase prototype of the PST is circularly symmetric about the frequency variable, the PST core phase shall be obtained:

(2) $$\begin{array}{c} \varphi \left(u,v\right)=\varphi_{\text{polar}}\left(r,\theta \right)=\varphi_{\text{polar}}\left(r\right), \end{array}$$

where *r* is the polar radius in the planar polar o-uv polar coordinate system of frequency domain; *θ* is the polar angle, and its relationship with the uv frequency variable is $r=\sqrt{u^{2}+v^{2}}$, *θ*= tan^−1^( *v*/*u* ). If it is required that the derivative of *φ* polar(*r*) with respect to *r* is an inverse tangent of the S-type, then

(3) $$\begin{array}{c} \frac{d\varphi_{\text{polar}}}{dr}=\;\tan^{-1}\left(r\right). \end{array}$$

*φ* polar(*r*): note that the uv frequency plane after the Fourier transform of the image is a finite region, then *φ*polar(*r*) can be solved according to

(4) $$\begin{array}{c} \varphi_{\text{polar}}\left(r\right)=\int_{0}^{r}\tan^{-1}\left(x\right)\text{d}x=x\cdot {\tan^{-1}\left(x\right)|}_{0}^{r}-\int_{0}^{r}\frac{x}{1+x^{2}}\text{d}x=r\;\tan^{-1}\;r-\frac{1}{2}\ln\left(1+r^{2}\right). \end{array}$$

The phase function in equation (4) was normalized to obtain *ϕ*_*N*_:

(5) $$\begin{array}{c} \varphi_{N}\left(r\right)=\frac{r\;\tan^{-1}\;r-\left(1/2.\right)\ln\left(1+r^{2}\right)}{r\;\tan^{-1}\;r\left(1/2\right)\ln\left(2_{\max\max\max}\right)}. \end{array}$$

For the phase function in equation (5), the phase tensile strength parameter *S* and the warping parameter *W* in the nonlinear distortion stretching transformation were added to obtain the final kernel phase function *ϕ*_*N*_(*r*, *W*, *S*) with strength parameter S and warping parameter in the PST transformation:

(6) $$\begin{array}{c} \varphi_{N}\left(\;\right.r\left.\;\right)=S\cdot \frac{Wr\;\tan^{-1}\left(\;\right.Wr\left.\;\right)-\left(1/2\right).\;\ln\left(\;\right.1+{\left(Wr\right)}^{2}\left.\;\right)}{r\;\tan^{-1}\left(1/2\right)\ln\left(\;\right.2_{\max\max\max}\left.\;\right)}, \end{array}$$

where tan-1(•) represents the inverse-tangent function, ln(•) is the natural logarithm, and rmax represents the maximum frequency polar radium of the uv frequency plane.

A PST having a kernel phase shaped as shown in equation (6) is applied to the spectrum of the digital image, forming a phase image *A*(*m*, *n*). For the application of the edge detection, subsequence operations can be implemented such as translation, thresholding, and morphological processing.

### 2.2. Fractional Order

#### 2.2.1. Basic Theory of Fractional Order

The Fractional Fourier Transform (FrFT) has more new unique features based on retaining the original properties of the traditional Fourier transform, so it can be considered to be a generalized Fourier transform [11].

In general, the *p*-order FRFT of the function *x*(*t*) can be expressed as *X*_*p*_(*u*) or *F*^*p*^ *x*(*t*) as needed. *F*^*p*^*x*(*t*) can be seen as an operator *F*^*p*^ acting on the signal *x*(*t*).

The basic definition of the FRFT was given below from the perspective of linear integral transformation. The *p*-order FRFT of the function *x*(*t*) in the time domain is defined as a linear integral operation:

(7) $$\begin{array}{c} X_{p}\left(u\right)=\int_{-\infty }^{+\infty }{\tilde{K}}_{p}\left(u,t\right)x\left(t\right)\text{d}t. \end{array}$$

Among them, ${\tilde{K}}_{p}\left(u,t\right)=A_{\alpha }\exp\left[j\pi \left(u^{2}\cot\;\alpha \;-\;2ut\;\csc\;\alpha +t^{2}\cot\;\alpha \right)\right]$ and ${\tilde{K}}_{p}\left(u,t\right)$ is called as the kernel function of the FRFT, where $A_{\alpha }=\sqrt{1-j\;\cot\;\alpha }$, *α*=*pπ*/2, *p* ≠ 2*n*, and *n* is an integer.

By variable substitution $u=u/\sqrt{2\pi }$ and $t=t/\sqrt{2\pi }$, equation (7) can be further expressed as

(8) $$\begin{array}{c} X_{p}\left(\;\right.u\left.\;\right)=\left\{\;\right.F^{p}\left[\;\right.x\left(\;\right.t\left.\;\right)\left.\;\right]\left.\;\right\}\left(\;\right.u\left.\;\right)=\int_{-\infty }^{+\infty }K_{p}\left(\;\right.u,t\left.\;\right)x\left(\;\right.t\left.\;\right)\text{d}t,\;0\;<\;\left|p\right|<2,0\;<\;\left|\alpha \right|<\pi \\ =\left\{\begin{array}{cc} B_{\alpha }\int_{-\infty }^{+\infty }\exp\left(j\frac{t^{2}+u^{2}}{2}\cot\;\alpha -\frac{jtu}{\sin\;\alpha }\right)x\left(t\right)\text{d}t, & \alpha \neq n\pi ,\\ x\left(t\right), & \alpha =2n\pi ,\\ x\left(-t\right), & \alpha =\left(2n+1\right)\pi . \end{array}\right. \end{array}$$

In equations (1)–(8), $B_{\alpha }=\sqrt{\left(1-j\;\cot\;\alpha \right)/2\pi }$; the linearity of FRFT given cannot indicate that this equation is unchanged, except for (*p*=4*n*), because the kernel function is not only the function of (*u*, *t*), but also that of order *p*. At *p* = 1, *α*=*π*/2, *A*_*α*_=1, and

(9) $$\begin{array}{c} X_{1}\left(u\right)=\int_{-\infty }^{\infty }x\left(t\right)e^{-j2\pi ut}\text{d}t. \end{array}$$

Thus, *X*_1_(*u*) is the common Fourier transform of *x*(*t*). Similarly, *X*_−1_(*u*) is the inverse transform of *x*(*t*) in the traditional Fourier transform. Because *α*=*pπ*/2 can only appear in the parameter position of the trigonometric function, the parameter *p* is defined by a period of 4, and it only needs to examine the interval *p* ∈ (  − 2,2 ]. At *p*=0, *f*_0_(*u*)=*f*(*u*); at *p*=±2, *f*_±2_(*u*)=*f*(−*u*).

All the above can be expressed as operators:

(10) $$\begin{array}{c} F^{0}=I,\\ F^{1}=F,\\ F^{2}=P,\\ F^{3}=FP=PF,\\ F^{4}=F^{0}=I,\\ F^{4n\pm p}=F^{4n^{\prime }\pm p}=F^{\pm p}, \end{array}$$

where *n*, *n*′ are the arbitrary integers.

The additivity of fractional order is a very important property of the FRFT, which can be expressed as

(11) $$\begin{array}{c} F^{p_{1}}F^{p_{2}}=F^{p_{1}+p_{2}}=F^{p_{2}}F^{p_{1}}. \end{array}$$

By using Gauss integrals to give direct integral representations, the operation shall be simpler, and then

(12) $$\begin{array}{c} \int K_{p_{2}}\left(u,u^{\prime }\right)K_{p_{1}}\left(u,u^{\prime }\right)du^{\prime }=K_{p_{1}+p_{2}}\left(u,t\right). \end{array}$$

In summary, the FRFT can be explained as follows: only considering the interval 0 ≤ *p* ≤ 1, the FRFT is the original function at *p*=0, and it is the ordinary Fourier transform at *p*=1; when *p* gradually changes from 0 to 1, its FRFT smoothly changes from the original function to the common Fourier transform.

The FRFT can also be interpreted in another way. That is, the FRFT is defined as a rotation transform of a time-frequency plane, and the FRFT of *p*-order is a linear canonical transformation defined by a transformation matrix. Transform matrix is given as

(13) $$\begin{array}{c} M=\left[\begin{array}{cc} A & B\\ C & D \end{array}\right]=\left[\begin{array}{cc} \cos\;\alpha & \sin\;\alpha \\ -\sin\;\alpha & \cos\;\alpha \end{array}\right]. \end{array}$$

Among them, *α*=*pπ*/2. According to the definition of Radon transform, in a plane along different lines (the distance between the line and the origin is *d*, and the direction angle is *α*), the line integral is made for *f*(*x*, *y*), and the obtained *F*(*d*, *α*) is the Radon transform of the function *f*. Then, the matrix can be regarded as the two-dimensional rotation matrix on the time-frequency plane.

**Figure 2:**
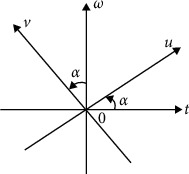
(*t*, *w*) plane rotating to (*u*, *v*) plane by *α* angle.

As shown in Figure 2, the Fourier transform can be considered as the representation of the function *x*(*t*) rotating the angle of *π*/2 from the *t* axis to the *ω* axis on the time-frequency plane; that is, the original function is mapped from the time domain to the frequency domain of the angle *π*/2 by Fourier transform; based on this, the FRFT is performed for the *p*_1_ and *p*_2_-order on the function, that is, with the fractional-order operator *F*^*p*_1_^ and *F*^*p*_2_^, the function is rotated by the *α*_1_ angle (*α*_1_=*p*_1_*π*/2) and the *α*_2_ angle (*α*_2_=*p*_2_*π*/2), respectively, and then mapped to *p*_1_and *p*_2_ orders.

#### 2.2.2. Discretization Method of Fractional Fourier Transform

The fast Fourier transform greatly promotes the development of the Fourier transform. Similarly, the fast FRFT algorithm will also rapidly develop the FRFT in the field of signal processing. Therefore, it is particularly important to study the discrete FRFT fast algorithm [12].

Ozaktas adopted the FRFT calculation method. This method performs N-point sampling on the time domain of the original function and also maps it to N sample points in the fractional Fourier domain. The computational complexity of the algorithm is O(N log N). Before using this method to calculate the FRFT, the original signal must be dimensionally normalized. Afterwards, in both the time and frequency domains, the representation of the signals is dimensionless and the length of support is equal to Δ*x*. This also indicates that the Wigner distribution of the signal is limited to the unit circle with Δ*x*/2 as the radius and the origin of the time-frequency plane as the centre. In order to obtain an efficient calculation method, the calculation of the FRFT is decomposed into a convolutional form. According to the definition of FRFT, the *a*-order FRFT of the signal *x*(*t*) can be written as

(14) $$\begin{array}{c} X_{\alpha }\left(u\right)=A_{\alpha }\;e^{-j1/2u^{2}\tan\left(\alpha /2\right)}\int_{-\infty }^{\infty }\left[x\left(t\right)e^{-j1/2t^{2}\tan\left(\alpha /2\right)}\right]e^{j1/2{\left(u-t\right)}^{2}\csc\;\alpha }\text{d}t. \end{array}$$

It can be seen from equations (1)–(13) that the calculation of the FRFT can be decomposed into three steps.

Step 1: the signal *x*(*t*) was multiplied by a chirp function, to obtain the intermediate result, which is recorded as *g*(*t*). Then, the frequency domain bandwidth of *g*(*t*) becomes 2 times more than that of the signal *x*(*t*), so the sampling interval of *g*(*t*) should be 1/2Δ*x*. The sampling interval of the original signal *x*(*t*) is 1/Δ*x*. At this time, if the sampling interval of *x*(*t*) is to be changed into 1/2Δ*x*, the signal *x*(*t*) needs to be interpolated twice to obtain the signal *x*(*t*) at the sampling interval 1/2Δ*x* and then multiply by the intermediate signal *g*(*t*) at the sampling interval 1/2Δ*x*.
Step 2: the signal *g*(*t*) is convolved with a chirp signal, because the bandwidth of *g*(*t*) is 2Δ*x*, so according to the convolution theorem, the chirp signal can be represented by its band-limited form 2Δ*x*, denoted as *h*(*t*):
  (15) $$\begin{array}{c} h\left(t\right)=\int_{-\Delta x}^{\Delta x}H\left(\Omega \right)e^{j\Omega t}\text{d}\Omega , \end{array}$$
where *H*(Ω) is the Fourier transform of the convolved chirp signal.
The convolution is written in discrete forms:
  (16) $$\begin{array}{c} g^{\prime }\left(\frac{m}{2\Delta x}\right)=\sum_{n=-N}^{N}h\left(\frac{m-n}{2\Delta x}\right)g\left(\frac{n}{2\Delta x}\right). \end{array}$$
This convolution formula can be calculated using the Fast Fourier Transform algorithm (FFT).
Step 3: multiply another chirp signal, to obtain the 2*N* sample points of *X*_*α*_(*u*) at the sampling interval of 1/2Δ*x*. Because it is a mapping of N sampling points in the time domain to N sampling points in the fractional Fourier domain, the *X*_*α*_(*u*)sampling at the sampling interval 1/Δ*x* can be obtained by performing a double extraction.

Let *X*_*α*_ and *x* be the column vectors consisting of N sample points of *X*_*α*_(*u*)and x(t), respectively; then, the above calculation process can be written in matrix form:

(17) $$\begin{array}{c} X_{\alpha }=F_{I}^{a}x,\\ F_{I}^{a}=D\Lambda \;H\Lambda J, \end{array}$$

where *D* and *J* represent the extraction and interpolation operations and Λ and *H* are the corresponding chirp multiplication and chirp convolution operations.

The method above expresses the FRFT as a convolution operation [13]. It is also possible to represent the FRFT in another form:

(18) $$\begin{array}{c} X_{\alpha }\left(u\right)=A_{\alpha }\;e^{j1/2u^{2}\left(\cot\;\alpha -\csc\;\alpha \right)}\int_{-\infty }^{\infty }\left[x\left(t\right)e^{j1/2t^{2}\left(\cot\;\alpha -\csc\;\alpha \right)}\right]e^{j1/2{\left(u-t\right)}^{2}\csc\;\alpha }\text{d}t. \end{array}$$

Sampling equations (2)–(13), it is calculated as

(19) $$\begin{array}{c} X_{\alpha }\left(\frac{m}{2\Delta x}\right)=A_{\alpha }e^{j1/2{\left(m/2\Delta x\right)}^{2}\left(\cot\;\alpha -\csc\;\alpha \right)}\sum_{n=-N}^{N}\left[x\left(\frac{n}{2\Delta x}\right)e^{j1/2{\left(n/2\Delta x\right)}^{2}\left(\cot\;\alpha -\csc\;\alpha \right)}\right]e^{j1/2{\left(m-n|/|2\Delta x\right)}^{2}\csc\;\alpha }. \end{array}$$

The summation of equation (18) can be expressed as a convolutional form of the signal, which was calculated using FFT. Finally, a 2*x* extraction was performed to obtain the *X*_*α*_(*u*) sampling at a sampling interval of 1/Δ*x*. Similarly, the above sampling process can be expressed in matrix form as

(20) $$\begin{array}{c} X_{\alpha }=F_{∐}^{a}x,\\ F_{∐}^{a}=DK_{a}J,\\ K_{a}\left(m,n\right)=A_{\alpha }e^{j1/2{\left(m/2\Delta x\right)}^{2}\left(\cot\;\alpha -\csc\;\alpha \right)-j1/2{\left(n/2\Delta x\right)}^{2}\left(\cot\;\alpha -\csc\;\alpha \right)+j1/2{\left(m-n|/|2\Delta x\right)}^{2}\csc\;\alpha }. \end{array}$$

## 3. Theoretical Analysis

### 3.1. Fractional-Order-Based PST

#### 3.1.1. PST Improvement Based on Fractional Order

It has been proven that the PST phase helps in detecting edges in the image as well as dramatic changes in intensity values [14]. However, edge detection for low image variations is less than ideal.

In image enhancement, the fractional differential can enhance the high-frequency component while enhancing the mid-low frequency component. To a certain extent, it can also nonlinearly preserve the DC component of the image, making the image texture details clearer and overcoming the defect that the integer-order differential can weaken the low-frequency information. With the efforts of scholars, the fractional differential enhancement has achieved certain results. Yang Zhuzhong et al. [15] used the G-L fractional differential to construct the Tiansi differential operator for image enhancement and stated that the differential order between 0.4 and 0.6 has a better enhancement effect; when the order is too large, the noise is also enhanced. In order to pass more low-frequency information, Wu Ruifang et al. [16] improved the Tiansi operator template. Wang Weixing et al. [17] modified the template to 8 different directions for greatly enhancing the edge information. Zhang Yu et al. [18] proposed an adaptive fractional-order enhancement algorithm, which applies an exponential function to determine the fractional order. Chen Qingli et al. [19] put forward the fractional-order enhancement algorithm based on Caputo's definition, which lays a good foundation for our study.

To this end, the paper combines PST and fractional order to improve the stretching operator of PST. The improved mathematical model is given as

(21) $$\begin{array}{c} A\left(m,n\right)=∠\text{FRFT}\left\{-\tilde{K}\left(u,v\right)\cdot \tilde{L}\left(u,v\right)\cdot \text{FRFT}\left[B\left(m,n\right)\right]\right\}, \end{array}$$

where *A*(*m*, *n*) is the angle image, “∠” is the angle operation, *B*(*m*, *n*) is the original input image, FRFT(^∗^) and FRFT(-^∗^), respectively, represent the fractional Fourier transform and Inverse transform, (*u*, *v*) is the frequency variable, $\tilde{L}\left(u,v\right)$ is the frequency response of the local smoothing filter, $\tilde{K}\left(u,v\right)=e^{j\phi \left(u,v\right)}$ is a frequency-dependent nonlinear phase warping kernel function, and *ϕ*(*u*, *v*) is a nonlinear function of the frequency variable.

### 3.2. Comparison and Analysis

It is assumed that the angle of the FRFT is *α*=*X*/*Y*, where *X* is the vector in the *X*-axis direction and *Y* is the vector in the *Y*-axis direction; the tensile strength parameter of PST is *S* and the warping parameter is *W*.

**Figure 3:**
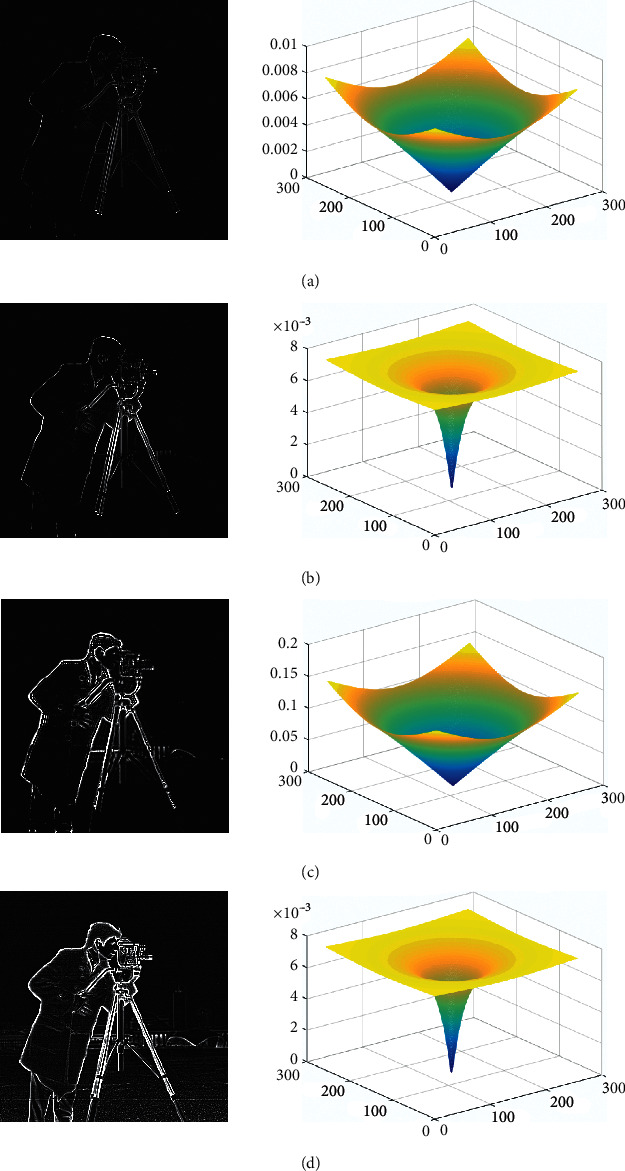
Comparison of edge detection test results under different values of (*X*) *Y*, *S*, and (*W*). (a) *X* = 0.5, *Y* = 0.5, *S* = 0.8, *W* = 1.0. (b) *X* = 0.8, *Y* = 0.5, *S* = 1, *W* = 15. (c) *X* = 0.8, *Y* = 0.8, *S* = 15, *W* = 1. (d) *X* = 1, *Y* = 1, *S* = 1.2, *W* = 15.

Then, the edge detection test results under different values of *X*, *y*, *s*, and *W* are compared, as shown in Figure 3.

### 3.3. Relative Total Variation (RTV) Analysis

To achieve the image enhancement effect, the extracted image from the edge and the original image need to be superimposed. The superimposed image highlights the edge features and achieves the enhanced result, but it may have noise, edge burrs, and so on. For this reason, a relative total variation (RTV) should be processed on the image to fuse its main and background images. The main principles of RTV are as follows.

To further enhance the contrast between texture and structure, especially for areas that are visually prominent, ℒ and ℋ were combined together to form a more efficient structural texture decomposition.

The objective function is assumed as

(22) $$\begin{array}{c} \arg\underset{s}{\min}\underset{p}{\sum }{\left(S_{p}-I_{p}\right)}^{2}+\lambda \cdot \left(\frac{{ℋ}_{x}\left(p\right)}{{ℒ}_{x}\left(p\right)+\varepsilon }+\frac{{ℋ}_{y}\left(p\right)}{{ℒ}_{y}\left(p\right)+\varepsilon }\right). \end{array}$$

Among them, (*S*_*p*_ − *I*_*p*_)^2^ ensures that there is no significant deviation between the input and the result. The introduction of the new regularization term ( ℋ_*x*_(*p*)/ℒ_*x*_(*p*)+*ε*+ℋ_*y*_(*p*)/ℒ_*y*_(*p*)+*ε* ) can achieve the effect of removing image texture, which is called relative total variation (RTV). *λ* in formula (22) is a weight; *ε* is a small positive number, avoiding being divided by zero.

The objective function in equation (22) is nonconvex. Therefore, its solution cannot be obtained in a common manner. Based on the quadratic measure penalty, the objective function was proposed, which is an effective solution method to make linear optimization (Szeliski, 2006; Lischinski et al., 2006; and Krishnan and Szeliski, 2011).

Our approach is to decompose RTV metrics into nonlinear terms and quadratic terms. Interestingly, the problem of the nonlinear part can be transformed into a series of linear equations by means of an iterative reweighted least squares method.

The metric in the *x* direction was firstly discussed and then that in *y* direction. The penalty term is expanded as

(23) $$\begin{array}{c} \sum_{p}\frac{{ℋ}_{x}\left(p\right)}{{ℒ}_{x}\left(p\right)+\varepsilon }=\sum_{p}\frac{\sum_{q\in R\left(p\right)}g_{p,q}\cdot \left|{\left(\partial_{x}S\right)}_{q}\right|}{\left|\sum_{q\in R\left(p\right)}g_{p,q}\cdot {\left(\partial_{x}S\right)}_{q}\right|+\varepsilon }. \end{array}$$

By regrouping the factor and the set element |(∂_*x*_*S*)_*q*_|, it is derived as

(24) $$\begin{array}{c} \sum_{p}\frac{{ℋ}_{x}\left(p\right)}{{ℒ}_{x}\left(p\right)+\varepsilon }=\sum_{q}\sum_{p\in R\left(q\right)}\frac{g_{p,q}}{\left|\sum_{q\in R\left(p\right)}g_{p,q}\cdot {\left(\partial_{x}S\right)}_{q}\right|+\varepsilon }\left|{\left(\partial_{x}S\right)}_{q}\right|\\ \approx \sum_{q}\sum_{p\in R\left(q\right)}\frac{g_{p,q}}{{ℒ}_{x}\left(p\right)+\varepsilon }\frac{1}{\left|{\left(\partial_{x}S\right)}_{q}\right|+\varepsilon_{s}}\\ =\sum_{q}u_{xq}w_{xq}{\left(\partial_{x}S\right)}_{q}^{2}. \end{array}$$

The second line is an approximation because *ε*_*s*_ for numerical stability was introduced. Through factor rearrangement, the metric was decomposed into a quadratic term (∂_*x*_*S*)_*q*_^2^  and a nonlinear part *u*_*xq*_*w*_*xq*_, which are expressed as

(25) $$\begin{array}{c} u_{xq}=\sum_{p\varepsilon R\left(q\right)}\frac{g_{p,q}}{{ℒ}_{x}\left(p\right)+\varepsilon }={\left(G_{\sigma }*\frac{1}{\left|G_{\sigma }*\partial_{x}S\right|+\varepsilon }\right)}_{q},\\ w_{xq}=\frac{1}{\left|{\left(\partial_{x}S\right)}_{q}\right|+\varepsilon_{s}}. \end{array}$$

Equation (25) indicates that *u*_*x*_ of each pixel actually merges adjacent gradient information in an isotropic spatial filtering manner; *G*_*σ*_ is a Gaussian filter with a standard deviation of *σ*. The division operation in (25) is performed by elements; ^∗^ is the convolution operator; and *w*_*x*_ is only related to the pixel gradient.

Similarly, the *y*direction penalty term can be expressed as

(26) $$\begin{array}{c} \sum_{p}\frac{{ℋ}_{y}\left(p\right)}{{ℒ}_{y}\left(p\right)+\varepsilon }=\sum_{q}u_{yq}w_{yq}{\left(\partial_{y}S\right)}_{q}^{2}. \end{array}$$

Among them, the secondary *y* component partial derivatives (∂_*y*_*S*)_*q*_^2^ and *u*_*yq*_*w*_*yq*_ are similar to the nonlinear part. They are respectively expressed as

(27) $$\begin{array}{c} u_{yq}=\sum_{p\varepsilon R\left(q\right)}\frac{g_{p,q}}{{ℒ}_{y}\left(p\right)+\varepsilon }={\left(G_{\sigma }*\frac{1}{\left|G_{\sigma }*\partial_{y}S\right|+\varepsilon }\right)}_{q},\\ w_{yq}=\frac{1}{\left|{\left(\partial_{y}S\right)}_{q}\right|+\varepsilon_{s}}. \end{array}$$

Through these operations, equation (22) can be written in matrix form:

(28) $$\begin{array}{c} {\left(v_{s}-v_{1}\right)}^{T}\left(v_{s}-v_{1}\right)+\lambda \left(v_{s}^{T}C_{x}^{T}U_{x}W_{x}C_{x}v_{s}+v_{s}^{T}C_{y}^{T}U_{y}W_{y}C_{y}v_{s}\right), \end{array}$$

where *v*_*s*_ and *v*_*I*_ are vector representations of *S* and *I*, respectively; *C*_*x*_ and *C*_*y*_ are Toeplitz matrices of the forward differential discrete gradient operators; and *U*_*x*_, *U*_*y*_, *W*_*x*_, and *W*_*y*_ are diagonal matrices. Their diagonal values are

(29) $$\begin{array}{c} U_{x}\left[i,i\right]=u_{xi},U_{y}\left[i,i\right]=u_{yi},W_{x}\left[i,i\right]=w_{xi},W_{y}\left[i,i\right]=w_{yi}. \end{array}$$

This makes it possible to achieve a special iterative optimization process. Due to the decomposition of the nonlinear part and the quadratic part, a numerically stable approximation is naturally obtained, which was found to be very effective in estimating fast structural and texture images in experiments.

## 4. Comparison of Test Results

**Figure 4:**
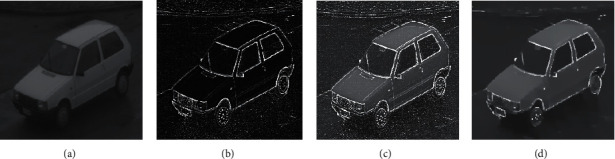
The flow chart of the experimental effect while *S* = 0.48, *W* = 12.24, *X* = 1, and *Y* = 1. (a) Original image. (b) Fractional PST edge extraction. (c) Superimposed image. (d) RTV processed image.

**Figure 5:**
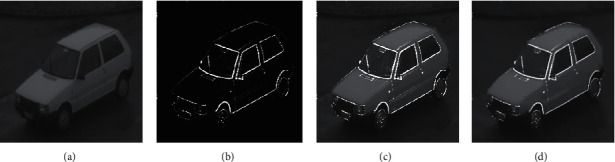
The flow chart of the experimental effect while *S* = 12.12, *W* = 0.50, *X* = 0.9, and *Y* = 1.1. (a) Original image. (b) Fractional PST edge extraction. (c) Superimposed image. (d) RTV processed image.

**Figure 6:**
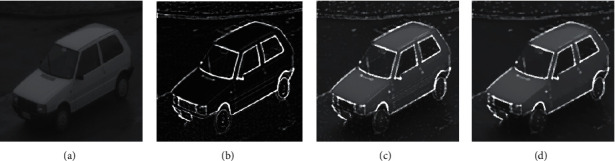
The flow chart of the experimental effect while *S* = 13.12, *W* = 13.14, *X* = 1, and *Y* = 1. (a) Original image. (b) Fractional PST edge extraction. (c) Superimposed image. (d) RTV processed image.

**Figure 7:**
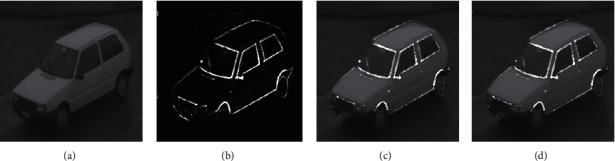
The flow chart of the experimental effect while *S* = 13.12, *W* = 13.12, *X* = 1.05, and *Y* = 0.95. (a) Original image. (b) Fractional PST edge extraction. (c) Superimposed image. (d) RTV processed image.

In this article, the test is divided into two parts. In the first part, the test data is based on tensile strength parameter *S* and the warping parameter *W* of the fractional PST takes different values. Let the angle of the FRFT be *α*=*X*/*Y*, where *X* is the vector in the *X*-axis direction, and the vector in which *Y* is in the *Y*-axis direction takes different values; the smoothness parameter Lam and the texture element parameter Sig of the RTV take different values. Taking multiple sets of images as test objects, the specific test results obtained are shown in Figures 4–7 and Table 1.

For the above test results, it is found that the tensile strength parameter *S* and the warping parameter *W* of the fractional PST are close to 0.5 and 10, respectively, and the angle of the FRFT is *α*=*X*/*Y*; when the values of *X* and *Y* are closer to 1, more edge information can be obtained, but more noise is added; when the smoothness parameter Lam and the texture size parameter Sig of the RTV are larger, the smoothing effect will be better, but the important edge information of the image will be blurred.

**Figure 8:**
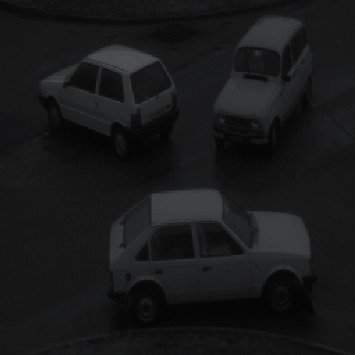
Original image 8. For the original image 8, the enhancement effects of the various algorithms are as follows.

**Figure 9:**
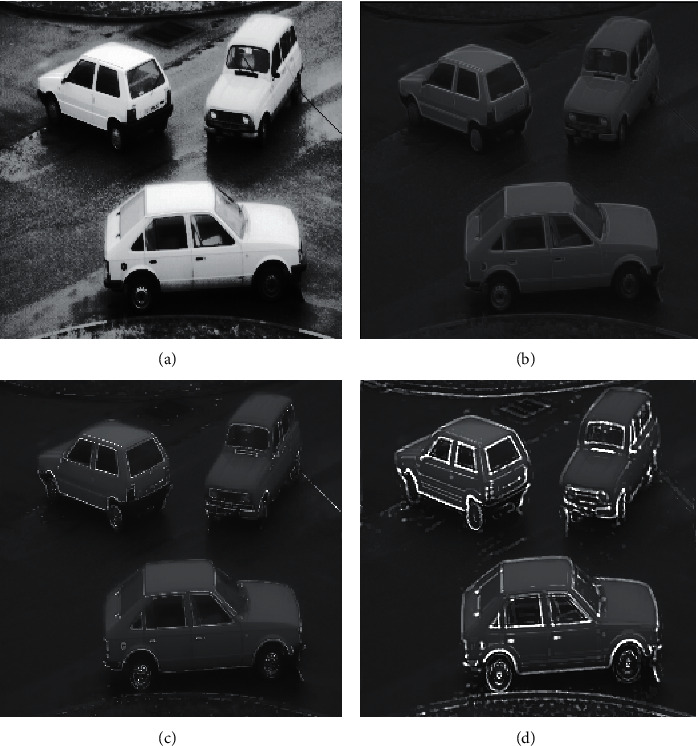
Enhancement effects of various algorithms. (a) Region growing-based image enhancement. (b) Operator-based image enhancement. (c) PST-based image enhancement. (d) Fractional-order PST-based image enhancement.

In the second part of the test, a comparative analysis was performed on image enhancement based on the fractional-order PST, phase consistency, region growing, histogram equalization, Canny operator, and so forth, respectively. The specific test results are shown in Figures 8 and 9.

The results of the above test show that some image enhancement algorithms such as the region growing-based image enhancement algorithm do not highlight the edge information while enhancing the high-frequency information of the image, and the resulting image will mask some important information; the image enhancement algorithms based on the canny operator and so forth may blur small edge information while presenting the edge portions of the image; the phase consistency-based edge enhancement algorithm can add edge burr or noise while highlighting edge information.

## 5. Conclusions

This article proposed a novel image enhancement algorithm, called FOPSTRTV to improve the quality of the image. Using the proposed algorithm to process the image, the noise in the original image is first eliminated by low-pass filtering. Then, the edge of the image is extracted through the differential order PST because the differential order PST processing can help to get the phase information and extract the image edge information well; in contrast, the edge extraction of the ordinary image is based on the time domain and frequency domain information. Finally, RTV can be used to fuse the image, giving a good visual effect. Extensive experiments with several representative algorithms demonstrated that FOPSTRTV is competitive and promising.

## Figures and Tables

**Table 1:** Comparison of the test data of different images.

Image	*S*	*W*	*X*	*Y*	Lam	Sig
Image 1	0.48	12.24	1	1	0.01	3
Image 2	12.12	0.50	0.9	1.1	0.008	2
Image 3	13.12	13.14	1	1	0.005	1
Image 4	13.12	13.12	1.05	0.95	0.001	1

## Data Availability

The data are available online at https://github.com/sunwww168/PSTRTV.
